# Teaching raters to see: A faculty development innovation for robust assessment in health professions education

**DOI:** 10.1186/s41077-026-00439-6

**Published:** 2026-05-05

**Authors:** Lon Setnik, Yoon Soo Park, Clément Buleon, Demian Szyld, Jenny Rudolph

**Affiliations:** 1 Center for Medical Simulation, 100 First Avenue Suite 400, Boston, MA 02129 USA; 2 Department of Medical Educatio, University of Illinois College of Medicine, 808 South Wood Street, Chicago, IL 60612 USA; 3 Center for Medical Simulation of Liège, University and University Hospital of Liège, Liège, Belgium; 4 Center for Medical Simulation of Normandie, NorSimS, University Hospital of Caen, Caen, France; 5 Department of Emergency Medicine, Boston Medical Center and Boston University Chobanian & Avedisian School of Medicine, 725 Albany Street, Boston, MA 02118 USA; 6 Harvard Medical School and Department of Anesthesia Critical Care and Pain Medicine, Beth Israel Deaconess Medical Center, Boston, USA

**Keywords:** Rater training, Staged learning, Debriefing, Medical education assessment, Digital rater training, Faculty development, Debriefing assessment, Competency-based education, Online training

## Abstract

Trustworthy simulation-based assessment depends on capable raters—but current approaches to rater training are often resource-intensive, inconsistently effective, and conceptually muddled. In practice, many programs focus on delivering training activities (e.g., frame-of-reference or behavioral observation training) without a clear model of the steps that build reliable, trustworthy assessments.

We clarify the pathway to reliable, trustworthy assessment skills by distinguishing rater cognition—the cognitive work of recognizing, interpreting, and judging performance—from rater training, the educational processes intended to develop that capability. Rating is not a mechanical act of applying a rubric; it is a skilled perceptual and interpretive process. As we argue, *a rubric does not do the seeing for you*. While rubrics externalize expert judgment, raters must be trained to recognize and apply that expertise—learning to distinguish observable data from inference and to “see through the eyes of the rubric.” Traditional rater training approaches can be reframed as mechanisms that build this underlying cognitive skill.

Building on this conceptual clarification, we introduce a staged learning framework for rater readiness. Rather than treating training as a one-time intervention, we propose that raters must be prepared developmentally: first through foundational deliberate practice, then through situational application, and finally through expert-facilitated calibration and ongoing feedback.

We illustrate this model through a pilot of an interactive, technology-enabled, partially asynchronous rater training program for the Advocacy–Inquiry Rubric. This pilot shows how simulation programs can greatly reduce the time required from experts to train raters, while also demonstrating feasibility, strong usability, and promising reliability outcomes.

## Introduction

Trustworthy simulation-based assessment depends on capable raters—yet current approaches to rater training are often resource-intensive, inconsistently effective, and conceptually diffuse. In practice, many programs emphasize delivering established training activities (e.g., frame-of-reference or behavioral observation training) without a clear model of how these activities build the capability required for reliable, trustworthy assessment.

In this paper, we shift the focus from rater training as an activity to rater readiness as an outcome. While the literature describes how to conduct rater training, organizations ultimately need raters who are prepared to perform in the real situations they will face. This reframing raises a set of practical questions: What assessment situations must raters be ready for? What are the fundamental skills of rating? What does readiness look like in a given context and for a given purpose? And how can organizations support ongoing learning beyond the initial training event—while leveraging technology to do so efficiently?

We begin by examining the current state of rater training and clarifying the relationship between rater training and rater cognition. We then introduce staged learning as a framework for developing rater readiness, using the analogy of training a code blue response team to ground the concept in familiar simulation practice. Finally, we illustrate this approach through a pilot implementation of a staged-learning rater training program for the Advocacy–Inquiry Rubric.

We conclude with lessons learned from this work and practical considerations for designing rater training systems that are effective, scalable, and responsive to emerging technologies.

### Reframing rater training: from techniques to cognitive skill development

Rater cognition and rater training are closely related but conceptually distinct. *Rater cognition* refers to the cognitive processes by which raters observe performance, interpret what they see, and integrate that information into judgments and ratings [1]. *Rater training*, in contrast, refers to the educational interventions designed to influence and develop those cognitive processes. Rater cognition is developed through rater training. Distinguishing between them clarifies both what is being developed and how.

Rating performance is more than applying a rubric or selecting a number on a scale. It involves a complex cognitive process in which raters must recognize relevant behaviors, process those behaviors through the interpretive lens of a rubric, and discriminate between different levels or qualities of performance. This cognitive work—often described as *rater cognition*—includes interdependent processes of observation, interpretation, and judgment that do not occur in a simple linear sequence but instead influence one another dynamically. Developing this capability requires training; it does not emerge automatically from exposure to a rubric alone.

Why use a rubric at all? Research on expertise in perceptual tasks suggests that while many of us have some working knowledge of a skill, that knowledge is often tacit, incomplete, and uneven—very different from the explicit, nuanced, and granular understanding held by experts [2]. Well-designed rubrics make that expert perception visible. They distill consensus across experts on what counts as excellence and its gradations [3, 4]. But a rubric does not do the seeing for you. Raters need training to recognize the expertise embedded in the rubric—and to apply it to what they observe.

One of the central goals of rater training, then, is to strengthen rater cognition. Strong rater cognition allows raters to answer crucial assessment questions quickly and reliably: What are the relevant behaviors I should be noticing? What do those behaviors mean in relation to the rubric’s standards? How would the rubric characterize the quality of this performance? And given all that, what rating is warranted? Getting to that level of fluency is not automatic. Raters must learn to move beyond their initial, often inference-laden impressions of a person or situation. A core skill is distinguishing between *data* (what was actually observed) and *inference* (what we think it means). Untrained judgment tends to collapse these together into a global gestalt. Training helps raters pull them apart—seeing behavior with greater precision while still forming an overall evaluation that is grounded, not assumed.

Rater cognition develops through the active construction of mental models and interpretive narratives about performance. Training methods aimed at helping raters “see through the eyes of the rubric” support this developmental process. “Seeing through the eyes of the rubric” is a metaphor we use to capture the intended endpoint of rater training: alignment between the rater’s internal mental models and the criterion-referenced standards articulated in the rubric. In this sense, *accuracy* refers to the degree of correspondence between raters’ evolving mental models and the explicit performance criteria [5, 6]. As rater cognition strengthens, unhelpful variability associated with novice or unstable judgment decreases, while meaningful differences among expert raters may persist. While not all variability is problematic, reducing unhelpful noise is particularly important in high-stakes assessment contexts [7].

Traditional rater training approaches in workplace-based assessment are well described and include *performance dimension training*, *behavioral observation training*, *and frame-of-reference training* [4, 8]. When viewed through a cognitive lens, these approaches can be understood as targeting different components of rater cognition. Performance dimension training supports perceptual attunement by helping raters recognize key behaviors through exposure to clear examples of effective and ineffective performance. Behavioral observation training develops the ability to bracket observations, selectively attend to relevant behaviors, and separate behavioral data from interpretive inferences. Frame of reference training integrates these skills by helping raters align their judgments with shared performance standards, supporting more consistent and criterion-referenced ratings. Together, these training approaches function as mechanisms for developing increasingly nuanced and reliable rater cognition.

The challenge, then, is not simply selecting the right training method, but organizing these methods into a developmental progression that builds rater readiness. Table 1 summarizes how specific rater training techniques align with rater cognition.

**Table 1:** How common rater training methods develop rater cognition

Step in Rater Cognition	What the Rater Must Do	Training Methods That Develop This Skill
Recognize	Identify and attend to relevant observable behaviors during a performance.	Performance Dimension Training (PDT) introduces raters to recognizable examples of different levels of performance. Typical activities include: • Reviewing observable examples of performance • Comparing effective and ineffective performance • Contrasting adjacent levels of performance quality Outcome: strengthens raters’ ability to recognize behaviors that correspond to rubric criteria and improves agreement on competency standards.
Process	Interpret observed behaviors in relation to rubric criteria and construct a judgment about performance quality.	Frame-of-Reference Training (FOR) aligns raters’ judgments with shared performance standards through: • Presentation and comparison of behavioral examples representing different effectiveness levels • Practice ratings followed by quantitative and qualitative feedback • Discussion of reasoning behind ratings Behavioral Observation Training (BOT) develops perceptual discipline through repeated opportunities to: • Select and bracket relevant behaviors • Distinguish behaviors from each other • Separate observable behaviors from interpretive inferences
Rate	Translate interpretations into consistent rubric-anchored judgments (e.g., narrative, numerical, or ranked ratings).	Frame-of-Reference Training (FOR) further supports this step by helping raters calibrate judgments to shared standards through repeated rating practice and comparison with expert benchmarks.

These approaches are often described separately in the literature. Here they are presented as complementary mechanisms for developing the cognitive processes involved in rating performance

### Staged learning

Rating is often treated as a static competency—something assessors either possess or acquire through brief exposure to a rubric or training session. We propose a different view: rating is a complex cognitive skill that develops over time. Like diagnostic reasoning, communication, or procedural expertise, rating can be deliberately trained through staged learning. We draw on Grow’s staged self-direction model [9] and Roussin and Weinstock’s SimZones framework [10] to outline a progression from foundational skill development to situational skill development, to systems promoting real-world readiness (Table 2).

**Table 2:** Rater training as staged learning with the example of Advocacy-Inquiry skill. Raters achieve “explicit expert-level knowledge of Advocacy-Inquiry skill” when they demonstrate readiness to select, process, and rate instances of Advocacy-Inquiry with consistency and clarity

Skill cluster	Foundational Rating Skills	Situational Rating Readiness	Expert-Facilitated Calibration Conversations
Method	Automated self-paced learning	Rating recordings with natural variability	Dialogue, reflection, and live coaching
Recognize and select behaviors	• Identify candidate behaviors for rating: – Assertions – Inquiries – Paraphrasing – Pauses/silences	• Apply recognition skills in real, messy recordings	• Identify inconsistencies in what gets selected for rating across cases or raters
Process and categorize behaviors	• Sort observed behaviors into rubric elements: – Preview – Observation – Point of View – Inquiry	• Sustain categorization when behaviors blur or overlap	• Clarify gray areas in categorization and align with peers or trainers on interpretations
Rate: apply rubric based judgment	• Apply criteria (Poor/Neutral/Good) to each element separately, then to all four elements together	• Rate full Advocacy-Inquiry sequences with natural variation and ambiguity	• Analyze causes of rating drift or inconsistency at individual or group level; develop shared interpretations and decision rules
Cognitive Discipline	• Separate observed behaviors from personal inferences or judgments	• Maintain this discipline under time pressure and cognitive load • Use the categories and scale of the rubric to mold narrative judgments	• Reflect on personal rating biases (e.g. hawk/dove, halo/horns, identity, personal values) or blind spots; surface tacit frames
Collective Practice and Calibration	--	--	• Compare and discuss divergent ratings • Co-create clarifying language or examples for future ratings • Contribute to community calibration routines

Staged learning is a process in which learners first develop foundational skills through instruction on how to execute a new skill. This instruction is aided by practice with feedback. Next, they situate those skills in important situations, this time with deliberate practice with feedback front and center. This deliberate practice includes “immediate feedback, time for problem-solving and evaluation, and opportunities for repeated performance to refine behavior [11].” Finally, expert facilitated conversations provide opportunities for trained people to discuss how to modify, maintain, or improve their performance in their context.

Code blue training with simulation offers a familiar example of staged learning: clinicians first master decontextualized foundational skills such as chest compressions with automated immediate feedback, they then practice those skills in context using methods such as rapid cycle deliberate practice, and finally integrate them within intact teams responding to complex team simulations or real events—continually learning through debriefs and outcome reviews. Preparing raters follows the same progression: foundational skills, situational practice, and real-world calibration.

### Staged learning for rater training

If rating is understood as a cognitive skill—one that involves perception, interpretation, and disciplined judgment—then rater training should be designed to develop that skill progressively. Raters first acquire foundational rating skills, then apply those skills in realistic assessment situations, and finally refine their judgments through calibration and ongoing learning in real-world contexts.

#### Foundational rating skill development

Foundational training develops the core components of rater cognition. Raters-in-training learn to recognize and bracket relevant behaviors, categorize those behaviors using the rubric, and evaluate performance quality using the rubric’s rating scale. Through deliberate practice—familiar to simulation educators through mastery learning [5] and rapid cycle deliberate practice [6]—raters repeatedly compare their judgments with expert benchmarks, gradually calibrating their internal standards.

Applied to rater training, foundational skill development includes:

- repeated rating of decontextualized examples designed to help learners recognize elements of the skill being assessed
- exposure to examples representing different performance levels
- clear performance criteria, such as behaviorally anchored descriptors
- structured practice rating fragments of performance using the rubric
- immediate, specific feedback comparing learner ratings to expert ratings
- gradually increasing complexity and nuance
- exposure to examples that combine multiple rubric elements

#### Situational rating skills with real world examples

Once foundational skills are established, training shifts toward situational readiness. In practice, raters evaluate performance in context, often under time pressure and with limited guidance. Situational training therefore places raters in realistic rating scenarios that resemble the situations they are likely to encounter in practice.

For example, the context for rating a communication skill such as delivering bad news may differ across assessment settings—such as an OSCE, a transition-to-practice program, or a fellowship evaluation. By constructing rating tasks that mimic these contexts, decontextualized foundational skills are applied in situations that more closely resemble real-world assessment.

Advancing to this stage without strong foundational skills increases extraneous cognitive load [12] and reduces rating consistency [8]. Similarly, without deliberate practice rating the types of encounters they will ultimately assess, raters may enter real-world evaluations without recognizing or regulating sources of bias or noise arising from their existing mental models [7, 13].

During situational skill development, ratings are strengthened through clear, criterion-referenced feedback driven by the rubric’s categories and rating scale. Structured comparison between trainee ratings and expert benchmarks supports calibration. Collecting both individual- and cohort-level data allows those training the raters to determine whether additional targeted situational training is warranted for the group as a whole or whether specific raters would benefit from further individualized development.

#### Expert facilitated calibration conversations

Rater readiness in practice involves more than technical skill. Ratings are influenced by identity, authority, entrenched patterns of perception, and emotional reactions [14] to observed behavior [13]. These factors are best addressed through expert-facilitated calibration conversations.

The bread and butter of these conversations is allowing raters to recognize, process, and rate a realistic example of performance and then discuss the narrative judgments behind their ratings with each other and with an expert rater. Here raters independently evaluate realistic examples of performance and then compare their reasoning with peers and expert raters. The goal is not only to reconcile rating differences but also to surface the assumptions, observations, interpretations, and emotions underlying those judgments. Through this process, raters refine their understanding of performance dimensions, clarify ambiguous cases, and strengthen their ability to distinguish observable behavior from interpretation.

Working collaboratively with facilitators and peers, raters identify barriers and facilitators that influence their judgments and develop strategies to support more consistent ratings. These conversations also build metacognitive awareness, helping raters recognize how their own perspectives, expectations, and emotional responses may shape their assessments.

#### Ongoing real-world rating feedback

Continuous readiness for rating situations should involve tracking real world outcomes with some regular meetings that explore what else has been learned as a result of the rating process and what adjustments might be needed in the rater training or rating process. Health professions schools, residency programs, and health system nursing development programs might invest in this activity as a strong link between professional development and the bedside readiness of their clinicians.

#### Summary of staged learning for rater training

By treating rater training as a skill that requires staged developmental training with timely feedback and, in the advanced stages, conversations that allow raters-in-training to surface and discuss their evolving mental models of performance, simulationists can apply the educational skills they already have from designing training programs to rater training programs.

### Our pilot: technology-supported staged learning to get raters ready to use the Advocacy-Inquiry Rubric

We next bring this framework to life through a modest pilot of a staged-learning rater training that uses technology to extend reach and reduce reliance on expert time. Presented as an innovation case in IMRD (Introduction, Methods, Results, Discussion) format, this example clarifies both the concept of staged learning and its practical application.

For clarity throughout, we use the following terminology: Advocacy-Inquiry refers to the conversational technique that we trained raters to assess; AIR refers to the Advocacy-Inquiry Rubric; and AI refers to generative artificial intelligence.

### Introduction: developing advocacy inquiry skills through rater training

Our goals were (1) to train peers or mentors to reliably assess the Advocacy-Inquiry conversation technique; (2) to build a rater training program that could combine the best of staged learning and rater training theories; (3) to reduce expert time required to develop skilled raters, and (4) to increase on-demand, flexible access for those who wanted to improve their rating skills, and (5) to determine next steps for rolling out similar rater training programs within our organization.

#### The advocacy inquiry rubric

Advocacy-Inquiry is a conversational technique employed by many debriefers to explore the actions of the students. Such exploration is a common phase in many forms of debriefings and learning conversations. The Advocacy Inquiry Rubric (AIR) was created to support training programs who want to provide feedback or formal rating on the Advocacy-Inquiry conversational technique, especially within simulation debriefings [15].

Since “Advocacy-Inquiry” was originally introduced in healthcare simulation [16], the authors and many other debriefing facilitators now add another type of speech [17], a “preview” or framing statement, before the Advocacy-Inquiry.

- Preview: The topic of the conversation
- Observation of behavior: The behavior that the conversation leader saw or heard the learner do
- “I think” statement: The impact of the observed behavior from the perspective of the debriefer
- Inquiry: A brief open-ended question to elicit the learner’s thought processes underlying the behavior

### Methods

We report on the structure of the pilot test of rater training as staged learning, then the quantitative and qualitative assessment of this pilot.

#### Research ethics review

This educational intervention project was submitted to the Mass General Brigham Human Research Office and was ruled exempt from continuing Institutional Review Board review.

#### Participants: raters-in-training

We piloted this training with three experienced debriefers known to the study team to gather focused feedback before broader deployment. All were expert debriefers with advanced training and experience but without prior exposure to the AIR. The group included a registered nurse with a doctorate (RN, BSN, PhD), an emergency physician and assistant director of a large academic simulation program (MD, MPH), and a speech pathologist who was a PhD candidate in Education. Each worked in U.S. health professions education institutions and had over 10 years of experience using and teaching Advocacy–Inquiry. We refer to them as the “raters-in-training.”

#### Foundational skills training

We started with an unusual combination: emoji ratings applied to an expert-derived rubric. The goal was not simplification, but clarity and reducing social awkwardness of numeric ratings. We wanted to train raters to see, interpret, and evaluate discrete elements of Advocacy–Inquiry with increasing accuracy, but in a way that was comfortable to use with peers.

To build these foundational skills—recognizing elements, processing them through the rubric, and assigning ratings—we developed an online, asynchronous active learning program using Genial.ly. Raters were presented with examples of each element (e.g., an isolated audio recording of a “Preview”), paired with the corresponding AIR element and an emoji-based rating scale. For foundational skills training each task was purposely simple: listen to the audio file, consult the rubric, select an emoji.

At its core, this design functions as rapid-cycle deliberate practice. Each rating attempt is immediately compared to an expert standard, making the gap between the learner’s judgment and the rubric explicit. Raters receive instant feedback indicating agreement or disagreement, along with a brief rationale, and can immediately revise their rating and try again. This tight loop—rate, compare, adjust—allows learners to calibrate their judgments in real time. As training progresses, examples become more nuanced, challenging the learner’s developing skill and sharpening perceptual discrimination.

Once raters demonstrated proficiency with isolated elements, they advanced to full Advocacy–Inquiry recordings. Here, they rated each element independently within a single interaction—four ratings per clip—with immediate feedback after each decision. The structure – hear a snippet, choose a rating, get feedback—remained consistent. But the cognitive demand increased.

The full training was asynchronous, required approximately two hours to complete, and was created, pilot-tested, revised, and then deployed.

Example images of each step of the training are provided in Table 3.

**Table 3:** Asynchronous rater training — automated rater training processes with embedded feedback

Rater Training Process	Online Asynchronous Active Learning Experience
Performance Dimension Training: A foundational activity that familiarizes raters with the definitions of different levels of performance. It groups similar levels of performance together or contrasts levels of performance to clarify rating criteria. Helps raters correctly associate performance examples with criteria. When doing competency-based assessment, performance dimension training enhances agreement on the criteria of competency	
Frame-of-Reference: Improves rating accuracy by entraining the users’ mental models to the “right” way of seeing things based on the rubric: • Presentation and discussion of multiple behavioral incidents representing different effectiveness levels • Practice rating followed by both quantitative and qualitative feedback Behavioral Observation Training: Repeated opportunities to select behaviors strengthening skill in: • Selecting and bracketing behaviors • Distinguish behaviors from each other • Distinguishing behavior from inferences	
Tips Pages	
Full Advocacy-Inquiry Ratings	

#### Situational skills rater training

The next step is to consolidate foundational skills by situating them in realistic assessment situations raters would likely confront in practice. In these situations raters do not evaluate isolated elements—they make judgments in context, sometimes under time pressure, and with minimal guidance [18].

The common use case: providing peer or mentor feedback on debriefing skills, a requirement in many accreditation and instructor development programs. To approximate this context, we used recordings of novice and intermediate debriefers practicing Advocacy–Inquiry statements at the end of a day of learning the technique, recordings that were realistic and variably executed.

Raters-in-training evaluated 22 audio clips representing a range of Advocacy–Inquiry attempts. Seventeen clips were drawn from actual training sessions; five were created to fill gaps in the performance spectrum. Clips ranged from 30 to 120 s, preserving enough context to require interpretation without overwhelming cognitive load.

Ratings were completed asynchronously using the GoREACT platform, which enabled systematic collection of ratings (Genial.ly did not support this function). This allowed us to examine inter-rater reliability across the cohort. Authors LS and JWR independently rated a subset of the clips to serve as a comparison standard, contributing to a total dataset of 760 ratings.

#### Expert facilitated calibration meetings

In the final phase of our staged learning model, training shifts from asynchronous to synchronous discussion and to more advanced work: metacognition about the rating process and refinement of raters-in-training’s understanding and application of rating criteria. These discussions strengthen the narrative judgments that support ratings, helping raters articulate not just *what* they rated, but *why*.

Anchored in areas of agreement and disagreement across ratings, these discussions used three data sources: the individual ratings of each rater-in-training, and the ratings of two expert raters (LS and JWR) applied to the same Advocacy–Inquiry recordings from Phase 2. Cases with the greatest discrepancy—either among raters or between raters and experts—were selected for discussion.

The goal of these conversations is not simply to resolve differences, but to analyze their sources. For example: were raters attending to different segments of behavior (e.g., identifying different phrases as the “preview”)? Were they applying different criteria (e.g., whether a preview must include time, place, and or a topic)? Or were they interpreting performance levels differently (e.g., what constitutes a “neutral” versus “smiley” rating)?

By making these distinctions explicit, raters refine both the granularity of their observations and the criteria underlying their judgments. These discussions help raters articulate not just *what* they rated, but *why*.

Through this process, raters moved toward shared interpretations of performance, achieving consensus on 20 of the 22 audio clips.

### Quantitative analysis

We sought evidence of reliability of the staged learning rater training process by measuring the agreement among raters using intraclass correlation. We explored the raters-in-trainings’ view of usability using the System Usability Scale.

### Qualitative analysis

To understand the subjective experience of using the scale we interviewed each expert rater, using a semi-structured interview (see Appendix 1). Semi-structured interview responses were analyzed using an inductive thematic analysis following Boyatzis [19] ^(p605)^. Interview data were first open coded to identify recurring concepts related to participants’ experiences with the training program. Codes were then grouped through iterative comparison into broader themes.

## Results

### Analysis of ratings

Intraclass Correlations (ICC) were calculated for the 760 ratings and reported in Table 4. We interpret this as high interrater consistency as measured by the intraclass correlation coefficient (ICC= 0.81) and good accuracy (agreement with expert rater’s gold standard) (ICC = 0.62), but due to the small numbers of participants in this pilot, the confidence intervals are wide, limiting the statistical conclusions we can draw [20]. The intraclass correlation, as evaluated by independent ratings of real-world examples, was calculated for exact agreement because our scale had only four possible ratings. The rating data did not follow a normal distribution, so Cohen’s Kappa was considered less valid for this data set [21]. Since the AIR was in development at the time, we did not have a “gold standard” set of ratings with which to compare these test ratings. Instead, we chose comparisons between each of the raters in the form of ICC. Both the participants and the authors assessed these real-world examples. The authors had extremely high agreement as demonstrated in Table 5.

**Table 4:** Rater consistency and accuracy by round: intraclass correlation

Item: Advocacy-Inquiry Rubric Element	Rater Consistency		Rater Accuracy	
	Round 1	Round 2	Round 1	Round 2
Preview	0.85	0.85	0.75	0.70
Observation	0.75	0.68	0.48	0.51
Point of View	0.86	0.84	0.65	0.76
Inquiry	0.83	0.83	0.47	0.66
Overall	0.82	0.80	0.59	0.66

Rater consistency describes the relationship between all the raters. Rater accuracy describes the relationship between the raters and the “gold standard” as set by the authors

**Table 5:** Pairwise intra-rater correlation

	Rater 1	Rater 2	Rater 3	Author 1	Author 2
Rater 1	1				
Rater 2	0.683178704	1			
Rater 3	0.514901005	0.701944599	1		
Author 1	0.599901664	0.499734402	0.264910189	1	
Author 2	0.677797186	0.500032554	0.236701776	0.911022999	1

The authors and two raters-in-training had excellent agreement in their ratings, but there was poor agreement between one assessor-in-training and their peers and the authors (Table 5).

While the size of our rater cohort limits our claims regarding generalizability, we also report usability by the participants and discuss what we can practically recommend based on our experience.

### Analysis of usability

We explored users’ experience with the system with a quantitative rating scale and qualitative semi-structured interview at the end of the entire process. We used the System Usability Scale [22, 23] an instrument used world-wide to measure perceived usability. The scale asks users to rate negative (e.g. unnecessarily complex) and positive (e.g. people could learn this system quickly) aspects of the system on a five-point agree/disagree measure. The SUS scores were 75, 80 and 95 points out of 100 as answered on the SUS with a standard deviation of 10.4 and a mean of 83.3. These scores are considered good (70–79) to excellent (80–100) [24].

### Analysis of the rater training experience

Three themes were identified through our thematic analysis: participants described increased clarity in understanding the rubric and debriefing concepts; they valued the flexibility of the asynchronous format for integrating the training into busy schedules; and they reported that synchronous consensus discussions supported reflection, calibration, and a sense of shared understanding among raters.

Raters-in-training endorsed the rater training program, as revealed by statements such as *“I feel like I learned a lot*,* [it] helped clarify hazy areas of debriefing”* and *“As we change the ways we educate our students*,* having people who are all on the same page will be vitally important. I think this work will expand in ways you have not imagined.”*

Raters-in-training additionally enjoyed the fact that they could schedule when and how they wanted. One rater-in-training performed the whole program in one sitting, while the others broke it up into smaller units. They would go back at times to look at sections that they wanted more time with. The raters-in-training described the program as fun, active, and useful.

Trainees were also enthusiastic about the consensus meetings; they experienced the dialogue and reflection included as a particularly rewarding part of the overall rater training program. They felt a sense of community with their new peers, and felt the conversation supported them in developing a nuanced view of the rubric. The following statement summarizes a representative user experience:

*“It works for adult learners. The convenience factor: if I have 15 minutes here or an hour there*,* I can squeeze it into my schedule. If we are trying to target people who are full time employees*,* they won’t have time to do things synchronously. The synchronous part was discussion*,* consensus*,* so I appreciated the asynchronous part that was preparation. [It] Allowed for the more valuable synchronous time*,* provided the open forum for discussion where they were needed and [did] not waste time where they weren’t needed.*

Overall, the feedback highlights that while the asynchronous flexibility was valuable at building cognition in the foundational skill training, and the social benefits to learning together in the [25] calibration meeting was also highly valued.

## Discussion

### Overview

The key contribution of this work is shifting the focus from delivering rater training activities to developing rater cognition through staged learning. Our pilot suggests that staged learning provides a coherent and practical framework for structuring rater training. We found that online, asynchronous foundational training is feasible, acceptable, and associated with improved inter-rater agreement, supporting the development of rater cognition.

In this conceptual innovation, we demonstrated an approach to training debriefing assessors that significantly reduces dependence on experts in rater training and shifts the burden to an interactive asynchronous rater training process for the foundational stage of rater training. Instead, we designed a staged learning process grounded in skill development, in which raters build capability through deliberate practice, contextual application, and calibration. Foundational skills training was effectively delivered through an asynchronous, online platform, enabling repeated exposure, immediate feedback, and progressive refinement of judgment.

Designing training to explicitly develop rater cognition helps raters learn to recognize, process, and evaluate behavior through structured comparison to expert standards. Assessors must be able to “see through the eyes of the rubric” to enact the goals of assessment. This work suggests that persistent challenges in rater variability may be addressed not only by refining rubrics, but by systematically developing the cognitive skills required to apply them. In this sense, rater training is not merely a technical exercise, but a mechanism for building shared mental models of performance among assessors. We propose that this approach represents a practical and scalable pathway for strengthening assessment in health professions education. This could be a helpful way to enhance workplace-based assessments for example the ACGME Milestones.

### Limitations

This study has several limitations related to design, measurement, and generalizability. First, our sample was limited in size and scope, including a small number of participants and a specific set of Advocacy–Inquiry recordings. We did not include pre-testing of participants’ baseline cognitive or rating abilities, which limits our ability to characterize learning gains. Additionally, the same recordings were used across training and reliability assessments, which may have introduced familiarity effects.

Second, the lower interrater agreement between one rater and others suggests that (1) rater training may not remove all variability; (2) that winnowing the pool of raters who consistently do not match other raters may be desirable in certain circumstances; or (3) capturing the wisdom of outlier raters in refining the next iteration of the rater training may be desirable. Indeed, emerging evidence in rater training continues to hone in on the elements that make it most effective, and the challenges in working with experts in health professions: “there may be faculty features limiting intended outcomes, such as imperviousness to rater training and difficulty resisting their own schemas.” [13]^(p1700)^.

Third, our measurement approach was constrained. We lacked a definitive gold standard for performance, and our statistical analyses were limited by the pilot nature of the project. While post-training agreement among raters was high, these findings should be interpreted as preliminary evidence of improved reliability rather than definitive validation.

Finally, this study focused on rating a conversational technique. Different domains of assessment may involve different tradeoffs in training design. For example, mastery learning approaches in procedural skills such as central venous catheter insertion may require alternative strategies for rubric implementation and rater preparation [26].

Despite these limitations, our findings provide early support for a staged learning approach to developing rater cognition and improving assessment reliability.

### Recommendations

#### Treat rater training as staged learning

Learning any complex skill follows recognizable developmental patterns, even as individual trajectories vary. Rater training can be strengthened by applying established principles of skill acquisition—such as deliberate practice and staged learning—to support progressive development from foundational skills to real-world readiness.

#### Break complex rating tasks into decontextualized elements early in training

To help raters “see through the eyes of the rubric,” we prioritized reducing cognitive load and isolating key elements of performance. By excerpting audio clips from broader contexts, raters were less influenced by impressions of the individual or situational factors—both known sources of bias in the initial stages of judgment. This design may support the development of more accurate, behaviorally anchored rating skills before reintroducing contextual complexity. Simplifying the task in early stages may accelerate skill acquisition, consistent with findings from other domains of learning.36.

#### Augment in-person training with asynchronous, feedback-rich learning

Asynchronous training that incorporates clear examples and immediate feedback can accelerate learning while reducing reliance on expert time. This approach enables scalable, high-frequency practice and allows expert effort to be concentrated where it is most impactful.

#### The role of AI in rater training

AI-driven approaches may accelerate and scale rater training, particularly in foundational skill development. AI systems could generate diverse performance examples, provide immediate feedback, and create high-volume opportunities for deliberate practice. In this way, AI could amplify the rapid-cycle learning we implemented in our Genial.ly platform while reducing the need for experts to create training materials and deliver feedback.

However, this efficiency introduces tradeoffs. In our approach, developing training examples—recording, labeling, and debating performance quality—also served as a mechanism for aligning expert judgment. AI-generated content may reduce this burden but may also bypass an important process of expert calibration.

AI and human raters offer complementary strengths. AI provides speed and scalability, while human raters bring contextual judgment and nuance. AI may therefore be most useful in foundational training, while later stages—particularly calibration discussions—continue to benefit from human expertise. Hybrid approaches combining AI practice with expert calibration will likely emerge.

## Conclusion

Rater training is often treated as a discrete activity; our findings suggest it is better understood as a process of skill development. By framing rating as a cognitive skill that can be built through staged learning, this work offers a practical approach to improving assessment in health professions education. Our pilot demonstrates that foundational rater cognition can be developed through scalable, asynchronous training with immediate feedback, which reduces the time demands on expert rater trainers, and that this learning can be extended through situational practice and expert-facilitated calibration.

This approach shifts the focus from refining rubrics alone to preparing raters to use them well. Developing the ability to “see through the eyes of the rubric” may be a critical step toward more consistent, meaningful, and trustworthy assessment. As assessment systems evolve—including the integration of AI—maintaining a focus on human judgment, shared standards, and ongoing calibration will remain essential.

## Data Availability

The data are available on request.

## Appendix 1: semi-structured interview questions

### Interview script

Thank you for participating in this program. We will take about 30-60 minutes to learn your thoughts. We have some ideas about themes to discuss, but this format can follow your interests as well.

#### Reactions

First, we would like to hear your initial reactions. How are you feeling right now about this program?

Based on their answers, we will draw from themes including learning, rating, relationships, and the process itself.

Possible questions included:

### Learning

Through your participation in this program, what did you learn?

What was the rating experience?

What did you learn about learning?

### What did you learn about online asynchronous learning?

#### Rating

What did you learn about rating?

How could we improve this process?

What else would you like us to know?

### Takeaways

Finally, we end with take-aways: what are you taking away from this program?
